# Modulation of Neutrophil Recruitment and Inflammatory Signaling in Acute Respiratory Distress Syndrome by Leukotriene Inhibitors Montelukast and Zileuton

**DOI:** 10.1096/fj.202501684R

**Published:** 2025-08-23

**Authors:** Anna Biedritzky, Yi Zhang, Anika Fuhr, Carolin Kleinmaier, Jutta Gamper‐Tsigaras, Ka‐Lin Heck‐Swain, Kristian‐Christos Ngamsri, Franziska Konrad, Michael Koeppen

**Affiliations:** ^1^ Department of Anesthesiology and Intensive Care Medicine University Hospital of Tuebingen Tübingen Germany; ^2^ Department of Anesthesiology Shenzhen Hospital of Southern Medical University Shenzhen Guangdong China; ^3^ Klinik für Anästhesiologie Orthopädische Klinik Markgröningen Markgröningen Germany

**Keywords:** acute respiratory distress syndrome, leukotriene inhibitors, montelukast, neutrophils, zileuton

## Abstract

Acute respiratory distress syndrome (ARDS) is characterized by excessive neutrophil‐driven inflammation and remains a leading cause of mortality in critical care. Leukotriene‐modifying agents, such as montelukast (a CysLTR1 antagonist) and zileuton (a 5‐lipoxygenase inhibitor), are approved for chronic inflammatory lung diseases, but their role in ARDS is unclear. We investigated the effects of montelukast and zileuton in a murine model of lipopolysaccharide (LPS)‐induced ARDS, supported by in vitro assays using human neutrophils. Mice were treated with either drug 1 h post‐injury. Neutrophil recruitment, cytokine release, and inflammatory signaling were assessed by immunohistochemistry, flow cytometry, ELISA, and qPCR. Neutrophil chemotaxis and signaling responses were evaluated in vitro. Both montelukast and zileuton significantly reduced neutrophil infiltration into lung tissue and bronchoalveolar lavage fluid (*p* < 0.01), suppressed expression of adhesion molecules (PSGL‐1, L‐selectin, LFA‐1), and decreased levels of TNF‐α, CXCL2, IL‐1β, and IL‐6 in BAL fluid (*p* < 0.05). In vitro, both drugs impaired neutrophil chemotaxis and reduced CysLTR1 and ERK1/2 expression following inflammatory stimulation. These findings indicate that leukotriene pathway inhibition limits neutrophil recruitment and activation in ARDS by modulating receptor expression and ERK1/2 signaling. Montelukast and zileuton may offer a targeted strategy to attenuate hyperinflammation in ARDS.

## Graphical abstract

Leukotriene inhibitors, montelukast and zileuton, modulate neutrophil recruitment and inflammatory signaling in acute lung injury. In a murine model of LPS‐induced acute lung injury, montelukast (a CysLTR1 antagonist) and zileuton (a 5‐lipoxygenase inhibitor) attenuate neutrophil transmigration into the alveolar space. This effect is mediated through (A) reduced recruitment of neutrophils to the alveolar compartment, (B) modulation of adhesion molecule expression (PSGL‐1, L‐selectin, and LFA‐1), (C) suppression of proinflammatory cytokines (TNF‐α, CXCL2, IL‐1β, IL‐6), and (D) downregulation of CysLTR1 and ERK1/2 signaling, which are essential components of leukotriene‐mediated inflammatory pathways.

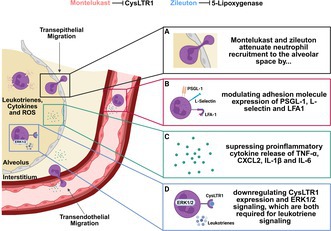

## 1 Introduction

Acute pulmonary inflammation frequently arises from an excessive immune response to harmful stimuli, including pathogens, toxins, and allergens. Although inflammation is essential for controlling infections and initiating tissue repair, an overactive response can lead to significant tissue damage, impaired lung function, and, in severe cases, life‐threatening conditions like acute respiratory distress syndrome (ARDS) [1, 2, 3]. In ARDS, this hyperinflammatory state is characterized by excessive infiltration of polymorphonuclear neutrophils (PMNs) and release of proinflammatory mediators, which increase microvascular permeability, promote edema, and impair gas exchange [4]. Current management strategies, including corticosteroids such as dexamethasone, aim to suppress this inflammatory response; clinical studies have demonstrated that reducing inflammation can lower mortality in COVID‐19‐associated ARDS [5]. However, corticosteroids broadly suppress immune activity, which increases the risk of secondary infections and other complications [1]. Interestingly, corticosteroids exert some anti‐inflammatory effects through inhibition of leukotriene synthesis, which limits neutrophil recruitment and leukotriene‐mediated inflammatory signaling [6].

Given the limitations of corticosteroids, there is a strong interest in alternative anti‐inflammatory therapies that selectively target key inflammatory pathways [7]. Leukotriene‐modifying drugs, such as montelukast and zileuton, offer a promising approach by specifically inhibiting leukotriene activity [8, 9, 10, 11]. Montelukast, a cysteinyl leukotriene receptor 1 (CysLTR1) antagonist, and zileuton, a 5‐lipoxygenase inhibitor, are widely used in chronic inflammatory diseases such as asthma, where they provide a targeted means to reduce inflammation without broad immunosuppressive effects [8, 12]. Leukotriene modifiers have shown anti‐inflammatory effects in several conditions, including liver disease, atherosclerosis, and rheumatoid arthritis [13, 14], suggesting broader potential applications in acute inflammatory settings.

While preliminary studies indicate that leukotriene modifiers may help reduce inflammation in models of acute lung injury, sepsis, and COVID‐19, little is known about their specific effects on PMN migration and the molecular mechanisms driving acute pulmonary inflammation [15, 16, 17]. Since PMN infiltration is a key contributor to lung injury in ARDS, understanding how leukotriene‐modifying drugs regulate this process could offer a targeted approach to mitigating hyperinflammatory responses in acute lung conditions.

To elucidate the mechanistic basis of leukotriene inhibition in ARDS, we focused on two key effectors of neutrophil function: the ERK1/2 branch of MAP kinase signaling and the surface expression of adhesion molecules. ERK1/2 kinases are activated downstream of immune receptors and chemokines and play an essential role in neutrophil activation, inflammatory gene expression, and cytoskeletal rearrangement [18]. Their activation links receptor‐mediated signals (e.g., TLRs, leukotriene receptors) to the cellular machinery that controls neutrophil migration, making ERK1/2 a critical node in neutrophil‐driven tissue inflammation. Notably, ERK1/2 activation is also required for leukotriene synthesis in neutrophils, suggesting a reciprocal amplification loop [19]. Adhesion molecules such as L‐selectin, PSGL‐1, and LFA‐1 mediate sequential steps in neutrophil recruitment: rolling, tethering, firm adhesion, and transendothelial migration [20, 21, 22]. Their dynamic regulation reflects neutrophil activation status and determines migratory efficiency toward inflamed tissues. We therefore selected these markers to investigate how leukotriene pathway inhibition modulates neutrophil trafficking and activation during acute lung inflammation. Our findings provide insight into the therapeutic effects of leukotriene inhibitors and may inform future strategies to manage excessive inflammation in ARDS and related acute lung injuries.

## 2 Materials and Methods

### 2.1 Animals

Male C57BL/6J wildtype mice, aged 8–12 weeks, were obtained from Charles River Laboratories (Sulzberg, Germany). All animal experiments were conducted in compliance with the guidelines of the Animal Care and Use Committee of the University of Tuebingen (A06/19G; Tuebingen, Germany) and adhered to the principles of the 3Rs (Replacement, Reduction, Refinement) to ensure ethical standards.

### 2.2 LPS‐Induced ARDS and Drug Administration

To induce ARDS, mice were exposed to lipopolysaccharide (LPS) inhalation (0.5 mg/mL dissolved in a total volume of 7 mL sterile saline, derived from *Salmonella enteritidis* , Sigma‐Aldrich, USA) in a custom‐built chamber. Nebulized LPS solution was delivered to each mouse, which resulted in a robust and reproducible pulmonary inflammatory response characterized by increased PMN migration into lung compartments and elevated microvascular permeability.

Montelukast (0.1 μg/μL at 1 μg/g body weight; Cayman Chemical, USA) and zileuton (0.1 μg/μL at 1 μg/g body weight; Cayman Chemical, USA) were administered intraperitoneally 1 h after LPS exposure [23]. Pre‐experiments established that post‐LPS administration yielded the most significant effects, guiding our choice of this time point for subsequent experiments to enhance clinical relevance.

### 2.3 Immunohistochemistry

PMN accumulation in the lung was assessed 24 h post‐LPS exposure, with or without montelukast or zileuton treatment. Mice were perfused to remove blood from the pulmonary circulation, and the lungs were fixed via tracheal cannulation with 4% paraformaldehyde (PFA) for 10 min at 25 cm H_2_O. The lungs were subsequently excised, fixed in 4% PFA for 24 h, and embedded in paraffin. Paraffin sections (3 μm) of lung tissue were processed for immunohistochemistry using the Vectastain ABC kit (Vector Laboratories, USA). Lung sections were blocked with Avidin solution (Vector Laboratories, USA) for 1 h. PMNs were stained with rat anti‐mouse Ly6G (clone RB6‐8C5, Abcam, UK) overnight at 4°C. Sections were incubated with biotinylated anti‐rabbit IgG (Vector Laboratories, USA) for 30 min, followed by an incubation with the 3,3′‐diaminobenzidine (DAB) substrate. Mayer's hemalaun solution (Sigma Aldrich, Germany) was used for tissue counterstaining. Stained tissue slides were analyzed with a Leitz DM IRB microscope from Leica using the AxioVision software version 4.8.2 (Carl Zeiss MicroImaging, Germany).

### 2.4 Flow Cytometry

A flow cytometry‐based in vivo migration assay was utilized to quantify PMN migration into different lung compartments: endothelial adherence, interstitial space, and intra‐alveolar space (Figure S1). PMNs adhering to the pulmonary endothelium were labeled by an allophycocyanin (APC)‐conjugated anti‐Ly6G antibody (clone 1A8; BioLegend, USA), which was intravascular (i.v.) injected into the tail vein directly before anesthesia. In deep anesthesia, a thoracotomy was followed by blood collection via the heart. Non‐adherent circulating PMNs were removed by flushing phosphate‐buffered saline (PBS) without calcium or magnesium through the pulmonary vasculature. Alveolar PMNs were collected via bronchoalveolar lavage (BAL). Blood, homogenized lung tissue, and BAL were incubated with PerCP‐conjugated anti‐CD45 (clone 30‐F11; BioLegend, USA) and PE‐Cy7‐conjugated anti‐Ly6G (clone 1A8; BioLegend, USA) antibodies. PMNs were classified by lung compartment: endothelial adherent PMNs were additionally marked with APC‐Ly6G via the i.v. injected antibody and therefore identified as Ly6G double‐positive (PerCP‐CD45+/PE‐Cy7‐Ly6G+/APC‐Ly6G+), interstitial PMNs as Ly6G simple‐positive (PerCP‐CD45+/PE‐Cy7‐Ly6G+/APC‐Ly6G‐), and intra‐alveolar PMNs in BAL as PerCP‐CD45+/PE‐Cy7‐Ly6G+.

For assessing the surface expression of adhesion molecules on PMNs in blood, lung, and BAL (Figure S2), additional antibodies were used: PE‐conjugated CD162 (PSGL‐1, clone 2PH1; BD Biosciences, USA), Pacific Blue‐conjugated CD62L (L‐selectin, clone MEL‐14; BioLegend, USA), and PE‐conjugated CD11a/CD18 (LFA‐1, clone H155‐78; BioLegend, USA).

### 2.5 Elisa

Levels of proinflammatory cytokines (TNF‐α, CXCL1, CXCL2, IL‐1β, and IL‐6) were quantified in BAL fluid 3 h after LPS exposure. An enzyme‐linked immunosorbent assay (ELISA; R&D Systems, USA) was performed according to the manufacturer's instructions. Protein levels of phospho‐ERK1/2 and total ERK1/2 were measured in homogenized murine lung tissue 3 h after LPS exposure by a semi‐quantitative ELISA (abcam, UK) following the manufacturer's specifications.

### 2.6 In Vitro Chemotaxis Assay

Human neutrophil migration was assessed using a live‐cell imaging μ‐Slide Chemotaxis assay (ibidi, Germany). Neutrophils were isolated from the whole blood of healthy volunteers using Percoll gradients and adjusted to a concentration of 4 × 10^6^ cells/mL. Cells were stained with CellTracker Green CMFDA (Thermo Fisher Scientific, USA) and pretreated with either montelukast (10 μM) or zileuton (10 μM) for 30 min. The collagen‐coated μ‐Slides were prepared to create a stable gradient of the chemoattractant N‐formylmethionyl‐leucyl‐phenylalanine (fMLP; 10 ng/mL) across the slide. For each condition, three independent experiments were conducted with two technical replicates. Live cell imaging was performed on a Leica Stellaris 8 confocal microscope, and neutrophil migration was analyzed with ImageJ's “manual tracking tool” plugin by tracking 20 randomized cells over 10 frames per experiment.

### 2.7 Immunofluorescence Staining

Paraffin‐embedded lung sections from wild‐type mice were fixed in 4% PFA, permeabilized with 1% Triton X‐100, and blocked with 5% bovine serum albumin (BSA) in PBS for 1 h. Lung sections were stained with primary antibodies targeting Ly6G (rat anti‐Ly6G, clone RB6‐8C5; Abcam, UK), CysLTR1 (rabbit anti‐CysLTR1, antibodies‐online GmbH; Germany), and ERK1/2 (rabbit anti‐ERK1/2; Cell Signaling Technologies, USA). Secondary antibodies included Alexa Fluor 647 goat anti‐rat IgG, Alexa Fluor 488 goat anti‐rabbit IgG, and Alexa Fluor 594 goat anti‐rabbit IgG (Thermo Fisher Scientific). Nuclei were counterstained with DAPI (Roti‐Mount FluorCare, Carl Roth, Karlsruhe, Germany). Images were captured on a Leica Stellaris 8 confocal microscope and analyzed using ImageJ.

### 2.8 Gene Expression

Total RNA was isolated from murine lung tissue using TRIzol (Invitrogen, Carlsbad, CA, USA) per the manufacturer's protocol. Complementary DNA (cDNA) synthesis was performed with the Bio‐Rad iScript kit (Bio‐Rad, Munich, Germany). Gene expression levels of ERK1 (MAPK3) and ERK2 (MAPK1) were measured by quantitative PCR (qPCR) with the following primers: MAPK3 (5′‐AGT CTC TGC CCT CGA AAA CC‐3′, 5′‐ACT GTG ATG CGC TTG TTT GG‐3′) and MAPK1 (5′‐AAT TGG TCA GGA CAA GGG CTC‐3′, 5′‐GAG TGG GTA AGC TGA GAC GG‐3′).

### 2.9 Statistical Analysis

Statistical analyses were performed using GraphPad Prism for Windows (Version 9.1, GraphPad Software Inc., San Diego, CA, USA). Data are presented as means ± standard deviation unless otherwise specified. Data were tested for outliers using an outlier test (Method ROUT, *Q* = 1%). The normality of data distribution was assessed using the D'Agostino & Pearson and Shapiro–Wilk tests. For comparisons between groups with normally distributed data, a one‐way analysis of variance (ANOVA) followed by the Bonferroni post hoc test was employed. For non‐normally distributed data, the Kruskal‐Wallis test was applied. All statistical tests were two‐tailed, with significance set at *p* ≤ 0.05.

## 3 Results

### 3.1 Montelukast and Zileuton Reduce PMN Transmigration Into the Bronchoalveolar Space

**FIGURE 1:**
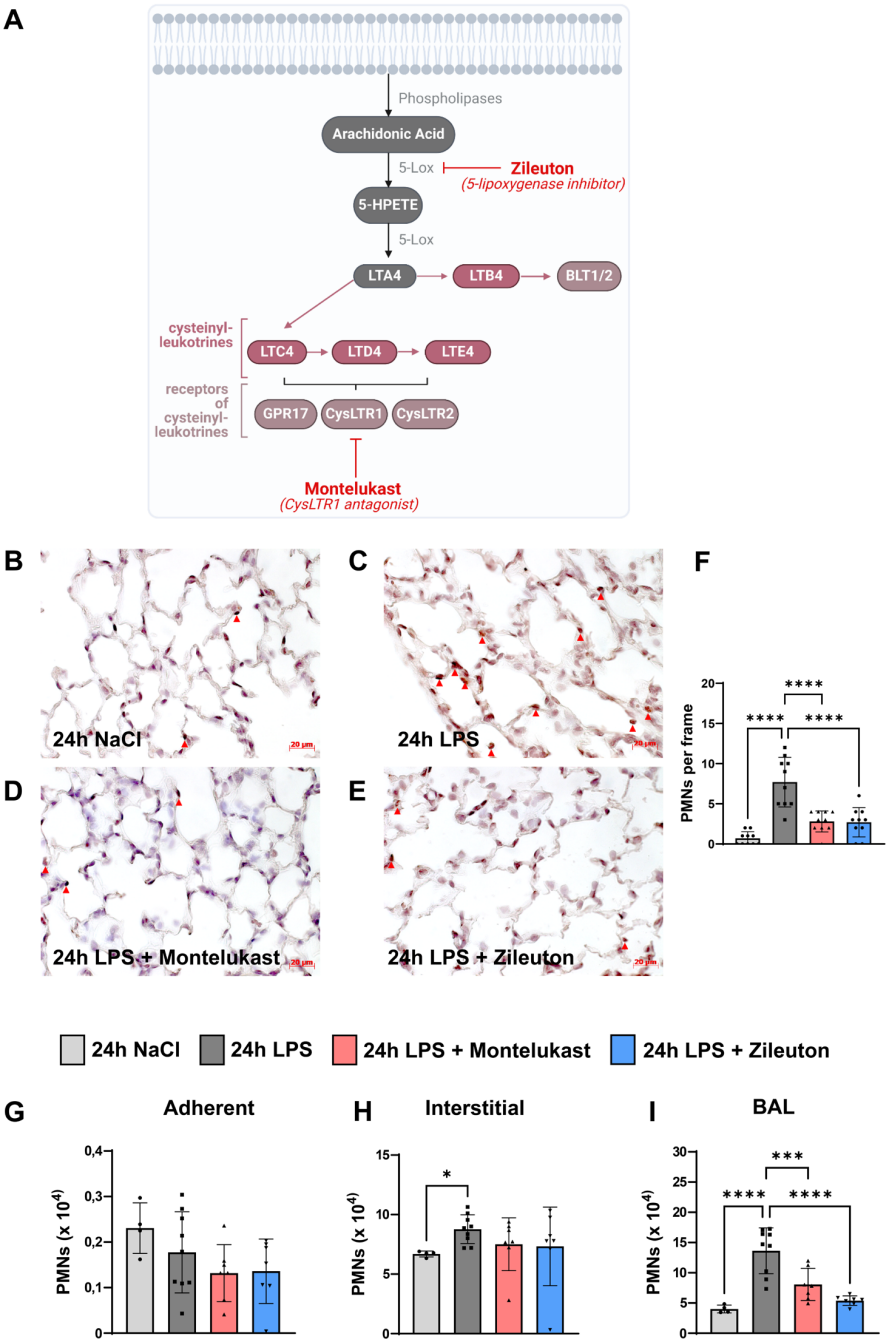
Reduced migration of PMNs into the lung with montelukast and zileuton treatment during acute pulmonary inflammation. (A) Schematic of the leukotriene synthesis pathway, illustrating the target sites of montelukast and zileuton. Created in BioRender. Koeppen, M. (2024) https://BioRender.com/c04r050. (B–E) Immunohistochemistry images showing PMN distribution in lung tissue under four conditions. PMNs were stained using an anti‐Ly6G antibody (brown), with hematoxylin counterstain. Red arrows indicate PMNs (representative images; *n* = 4 mice per group; ×63 magnification). (F) Quantification of PMN counts in high‐power fields (HPF; *n* = 4 mice per group, with 10 images counted per group; ×magnification). (G–I) Flow cytometry analysis of PMN distribution across lung compartments: Adherent (G), interstitial (H), and BAL (I) (*n* = 4–9 mice per condition). The bar graphs were labeled according to the following color scheme for the four experimental conditions: 24 h NaCl in light gray, 24 h LPS in dark gray, 24 h LPS + Montelukast in red, and 24 h LPS + Zileuton in blue. Data are presented as mean ± SD. Statistical analysis: *p < 0.05, ***p < 0.001, ****p < 0.0001, using one‐way ANOVA.

Leukotrienes are potent proinflammatory lipid mediators that promote neutrophil recruitment by enhancing endothelial adhesion and vascular permeability [24]. Montelukast, a CysLTR1 antagonist, and zileuton, a 5‐lipoxygenase inhibitor, target the leukotriene synthesis pathway at distinct points, with montelukast blocking CysLTR1 and zileuton preventing leukotriene formation (Figure 1A). The 24‐h time point was chosen based on prior studies showing that neutrophil transendothelial and transepithelial migration peaks around 12–24 h after LPS exposure, making it an optimal window to assess leukocyte infiltration and activation [25].

To assess their effects on neutrophil infiltration, we treated C57BL/6J mice with montelukast or zileuton 1 h after LPS inhalation. After 24 h, neutrophil counts were quantified by immunohistochemistry and flow cytometry. Both inhibitors significantly reduced neutrophil recruitment into pulmonary tissue compared to the LPS‐only group (Figure 1B–F); though neither affected adherent or interstitial neutrophils in the pulmonary parenchyma (Figure 1G,H). However, both drugs markedly reduced neutrophil counts in bronchoalveolar lavage (BAL) fluid (Figure 1I), suggesting that montelukast and zileuton limit neutrophil migration into the bronchoalveolar space by retaining them in the interstitial compartment.

### 3.2 Alterations in PMN Adhesion Molecule Expression by Montelukast and Zileuton

**FIGURE 2:**
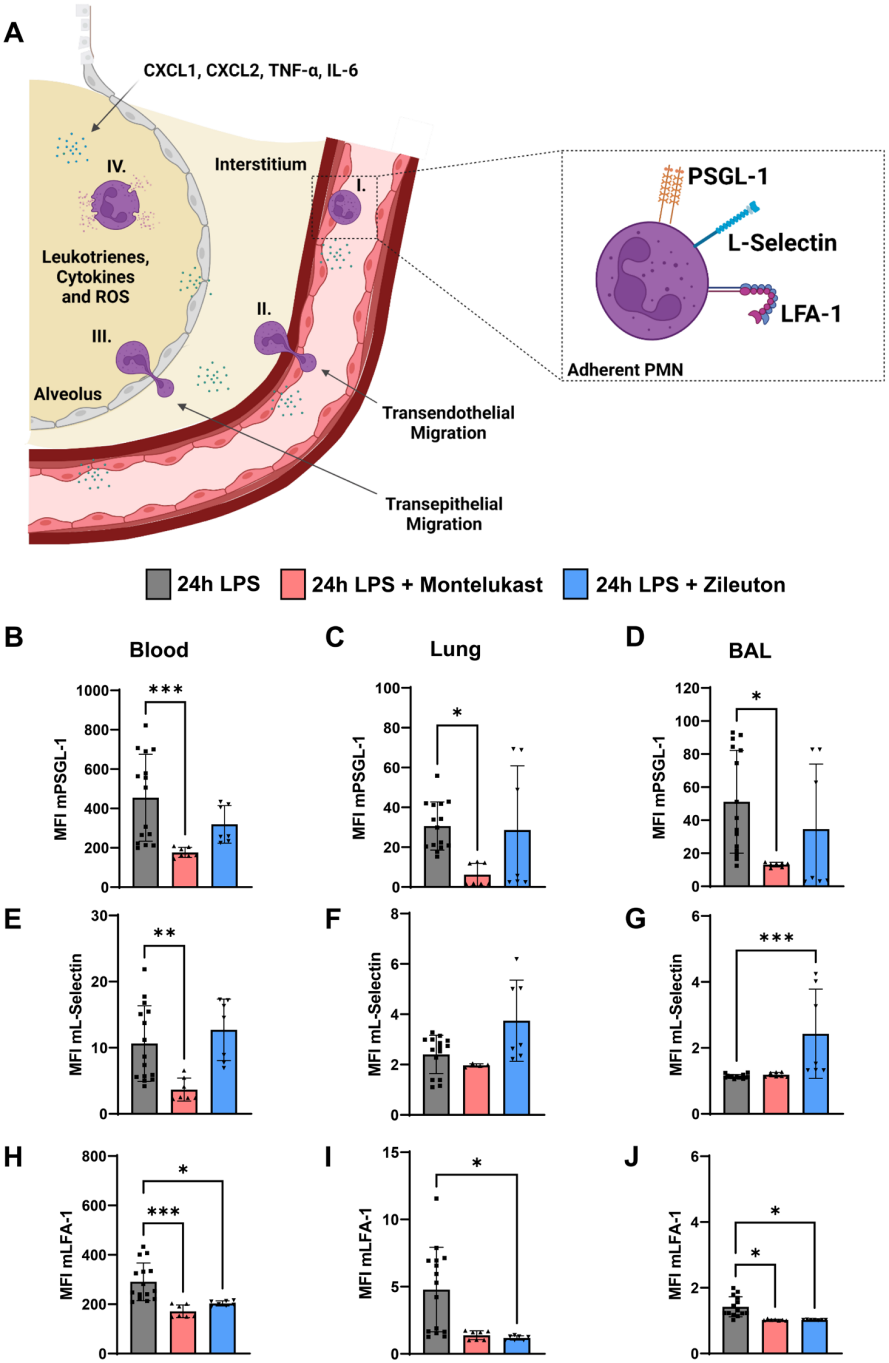
Montelukast and zileuton modulate PMN adhesion molecule expression. (A) Diagram of PMN migration from vasculature to alveolar spaces, highlighting key adhesion molecules: P‐selectin glycoprotein ligand‐1 (PSGL‐1), L‐selectin, and lymphocyte function‐associated antigen 1 (LFA‐1). Created in BioRender. Koeppen, M. (2024) https://BioRender.com/n68c366. (B–J) Mean fluorescence intensity (MFI) of PSGL‐1, L‐selectin, and LFA‐1 in blood, lung, and BAL 24 h post‐LPS (*n* = 7–15 mice per condition). The bar graphs were labeled according to the following color scheme for the four experimental conditions: 24 h NaCl in light gray, 24 h LPS in dark gray, 24 h LPS + Montelukast in red, and 24 h LPS + Zileuton in blue. Data are presented as mean ± SD. Statistical analysis: *p < 0.05, **p < 0.01, ***p < 0.001, using one‐way ANOVA.

To further investigate the mechanism underlying reduced PMN transmigration, we examined whether montelukast and zileuton alter neutrophil adhesion molecule expression. Neutrophils upregulate selectins and integrins to facilitate vascular adhesion and tissue migration (Figure 2A). We analyzed the expression of PSGL‐1, L‐selectin, and LFA‐1 on PMNs in blood, lung tissue, and BAL fluid using flow cytometry.

Montelukast significantly reduced PSGL‐1 expression in all compartments, whereas zileuton had no effect (Figure 2B–D). L‐selectin expression remained unchanged in lung tissue and BAL fluid, though montelukast reduced its levels in the bloodstream (Figure 2E–G). Both inhibitors decreased LFA‐1 expression on PMNs in the bloodstream and BAL fluid, with zileuton also reducing LFA‐1 in lung tissue (Figure 2H–J).

These findings indicate that montelukast and zileuton modulate specific adhesion molecules, potentially disrupting PMN interactions with endothelial and epithelial barriers and thereby reducing transmigration.

### 3.3 Reduction of Proinflammatory Cytokines in the Bronchoalveolar Space by Montelukast and Zileuton

**FIGURE 3:**
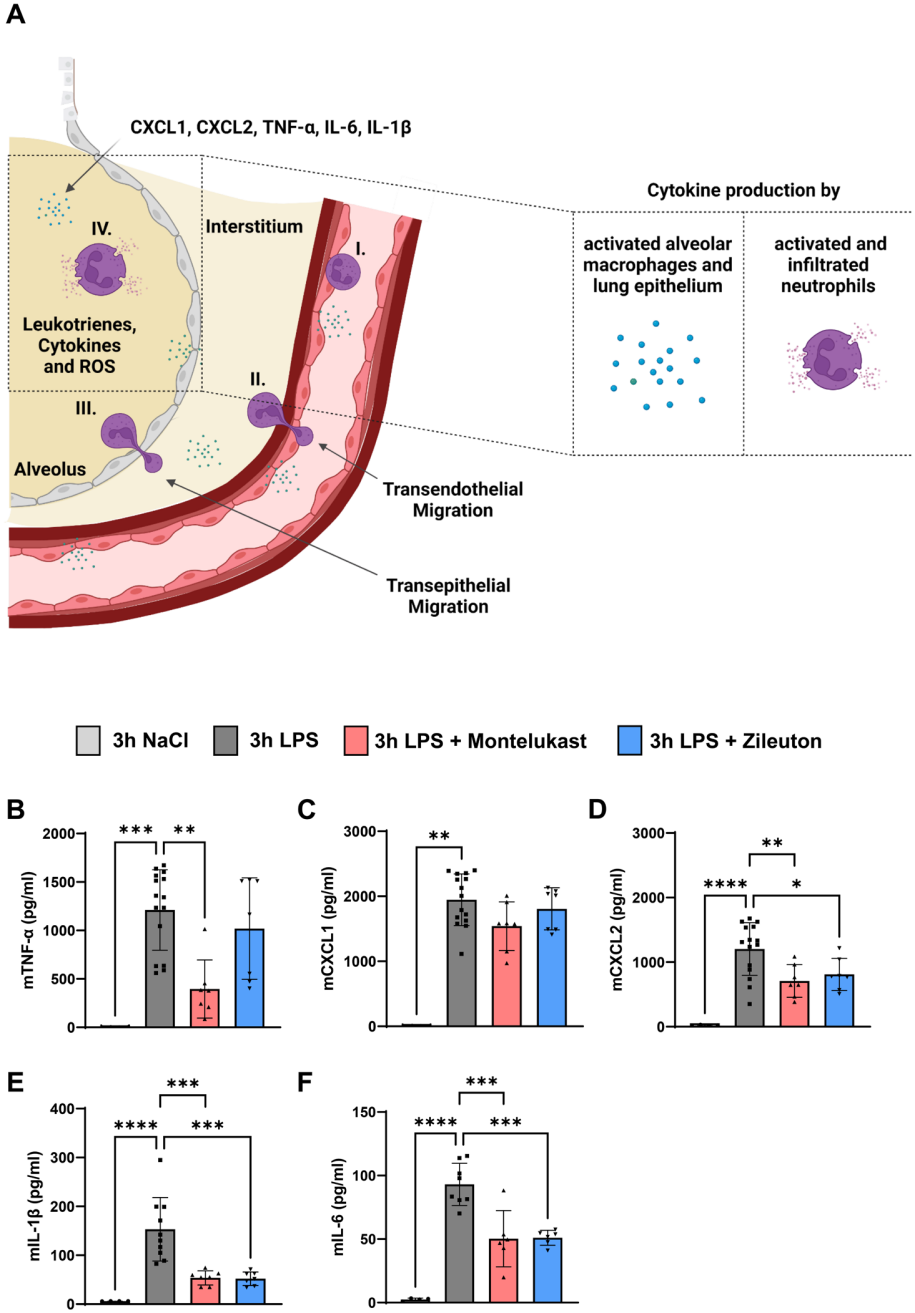
Montelukast and zileuton reduce proinflammatory cytokine release in BAL fluid. (A) Schematic of cytokine release from macrophages, epithelial cells, and infiltrated neutrophils promoting PMN migration. Created in BioRender. Koeppen, M. (2024) https://BioRender.com/r83r464. Protein levels of (B) TNF‐α, (C) CXCL1, (D) CXCL2, (E) IL‐1β, and (F) IL‐6 in BAL fluid were quantified 3 h after LPS exposure by ELISA (*n* = 4–15 mice per condition). The bar graphs were labeled according to the following color scheme for the four experimental conditions: 3 h NaCl in light gray, 3 h LPS in dark gray, 3 h LPS + Montelukast in red, and 3 h LPS + Zileuton in blue. Data are presented as mean ± SD. Statistical analysis: *p < 0.05, **p < 0.01, ***p < 0.001, ****p < 0.0001, using one‐way ANOVA.

Excessive cytokine release by alveolar macrophages and epithelial cells drives neutrophil recruitment and amplifies lung inflammation (Figure 3A). To determine whether montelukast and zileuton mitigate this response, we measured TNF‐α, CXCL1, CXCL2, IL‐1β, and IL‐6 levels in BAL fluid 3 h after LPS inhalation.

Both inhibitors significantly reduced CXCL2, IL‐1β, and IL‐6 levels (Figure 3C–F). Montelukast also suppressed TNF‐α, while zileuton had no effect on TNF‐α levels (Figure 3B).

These results suggest that montelukast and zileuton attenuate key cytokine signals that drive neutrophil infiltration into the inflamed lung.

### 3.4 Montelukast and Zileuton Inhibit PMN Chemotaxis In Vitro

**FIGURE 4:**
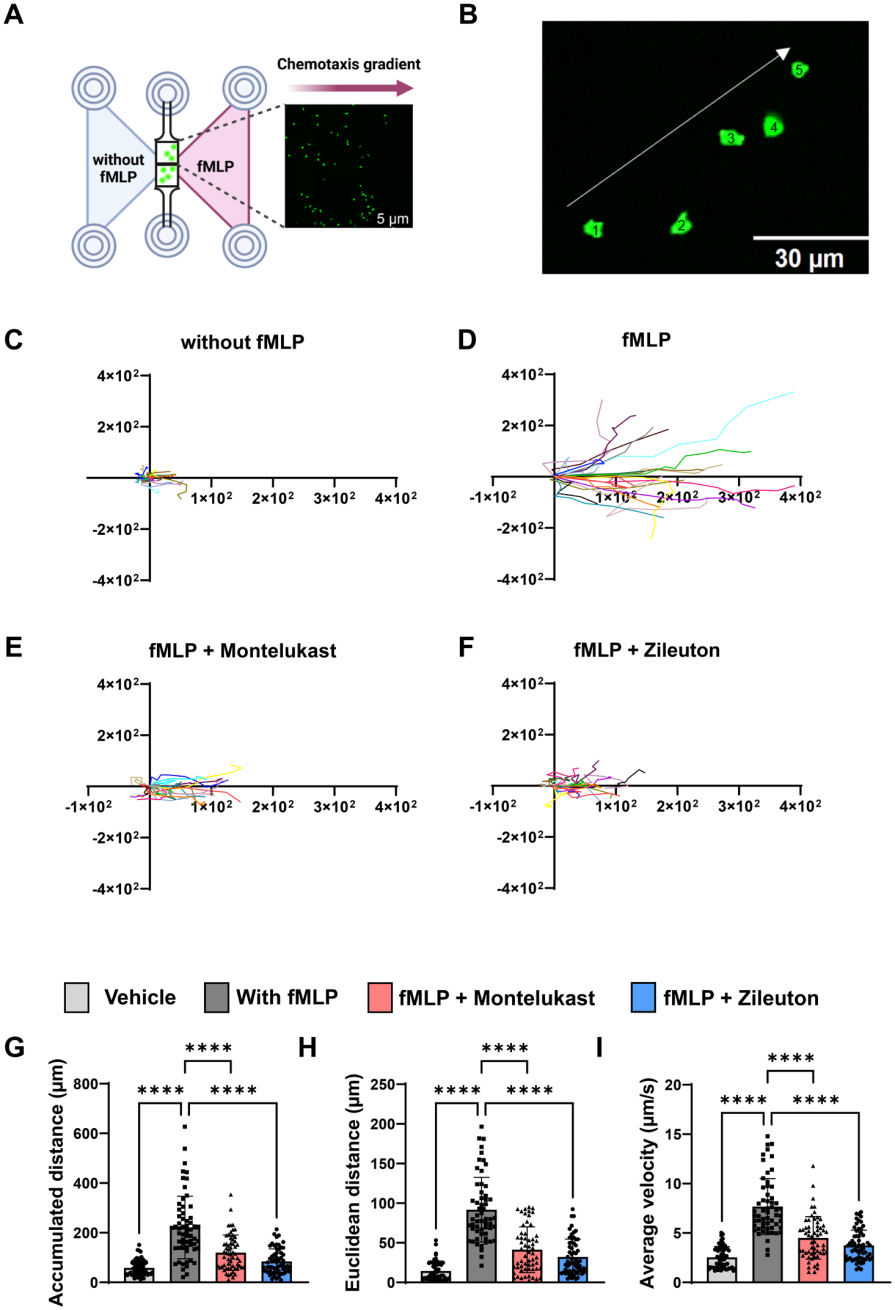
Montelukast and zileuton inhibit human PMN migration along an fMLP gradient in vitro. (A) Schematic of the μ‐Slide Chemotaxis assay. Created in BioRender. Koeppen, M. (2024) https://BioRender.com/m12s025. (B) Overlay images of a representative PMN's migration path over five frames. Migration of human PMNs under different conditions: (C) vehicle control, (D) fMLP, (E) fMLP + montelukast, and (F) fMLP + zileuton. (G–I) Quantification of migration parameters: accumulated distance (G), Euclidean distance (H), and average velocity (I) (*n* = 20 PMNs per condition across three experiments). The bar graphs were labeled according to the following color scheme for the four experimental conditions: vehicle in light gray, fMLP in dark gray, fMLP + montelukast in red, and fMLP + zileuton in blue. Data are presented as mean ± SD. Statistical analysis: ****p < 0.0001, using one‐way ANOVA.

To evaluate whether leukotriene pathway inhibition directly affects neutrophil chemotaxis, we performed an in vitro chemotaxis assay using an fMLP gradient, a potent neutrophil chemoattractant (Figure 4A,B). Untreated PMNs migrated robustly toward the gradient (Figure 4D); whereas montelukast and zileuton significantly reduced accumulated distance, Euclidean distance, and velocity (Figure 4E–I). These findings suggest that both inhibitors impair PMN chemotactic responsiveness, potentially contributing to their anti‐inflammatory effects in vivo.

### 3.5 Downregulation of Leukotriene Receptor Expression by Montelukast and Zileuton

**FIGURE 5:**
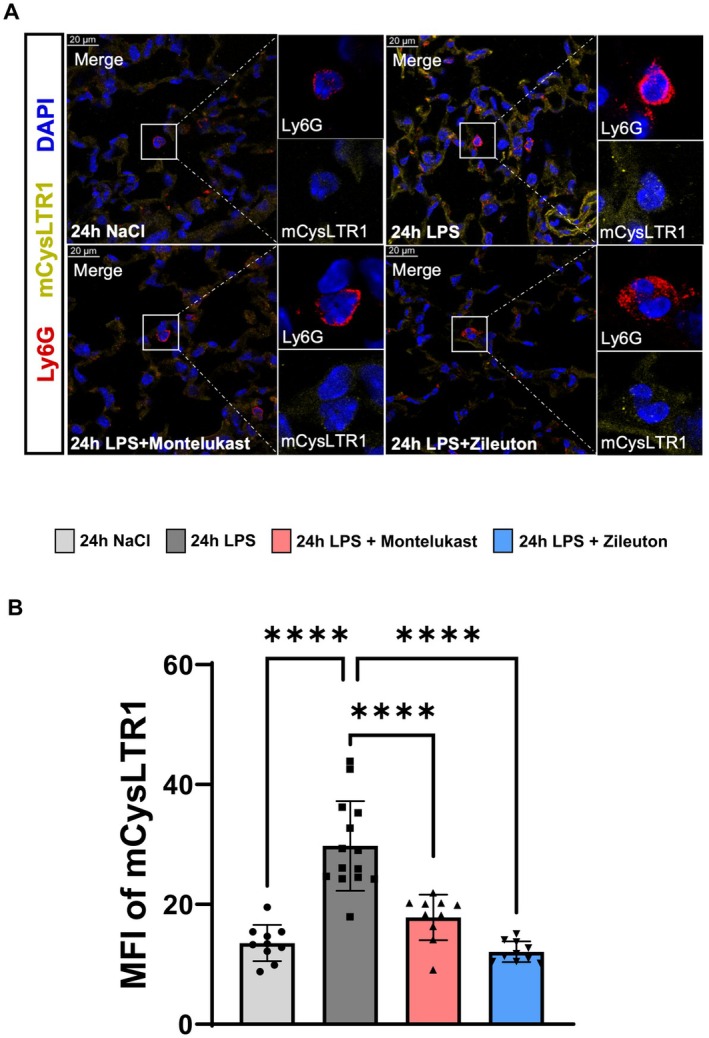
Montelukast and zileuton reduce CysLTR1 receptor expression on PMNs in murine lung tissue. (A) Representative immunofluorescence images showing CysLTR1 expression on PMNs in lung tissue (original magnification, ×63). (B) Quantification of CysLTR1 MFI using ImageJ (*n* = 4 mice per group; 10–14 measurements per group). The bar graphs were labeled according to the following color scheme for the four experimental conditions: 24 h NaCl in light gray, 24 h LPS in dark gray, 24 h LPS + Montelukast in red, and 24 h LPS + Zileuton in blue. Data are presented as mean ± SD. Statistical analysis: ****p < 0.0001, using one‐way ANOVA.

Since CysLTR1 mediates leukotriene signaling in neutrophils [6], we examined whether its expression is altered by montelukast or zileuton. Immunofluorescence staining of lung tissue revealed that both inhibitors significantly reduced CysLTR1 expression on PMNs in inflamed lung tissue (Figure 5A,B). This suggests that montelukast and zileuton not only block leukotriene signaling at the receptor level but also downregulate CysLTR1 expression, potentially dampening neutrophil responsiveness to inflammatory cues.

### 3.6 Montelukast and Zileuton Suppress the ERK1/2 Signaling Pathway

ERK signaling regulates neutrophil activation, chemotaxis, and inflammation. To assess whether montelukast and zileuton affect ERK signaling in pulmonary neutrophils, we quantified ERK1 (mMAPK3) and ERK2 (mMAPK1) expression in LPS‐treated mice.

**FIGURE 6:**
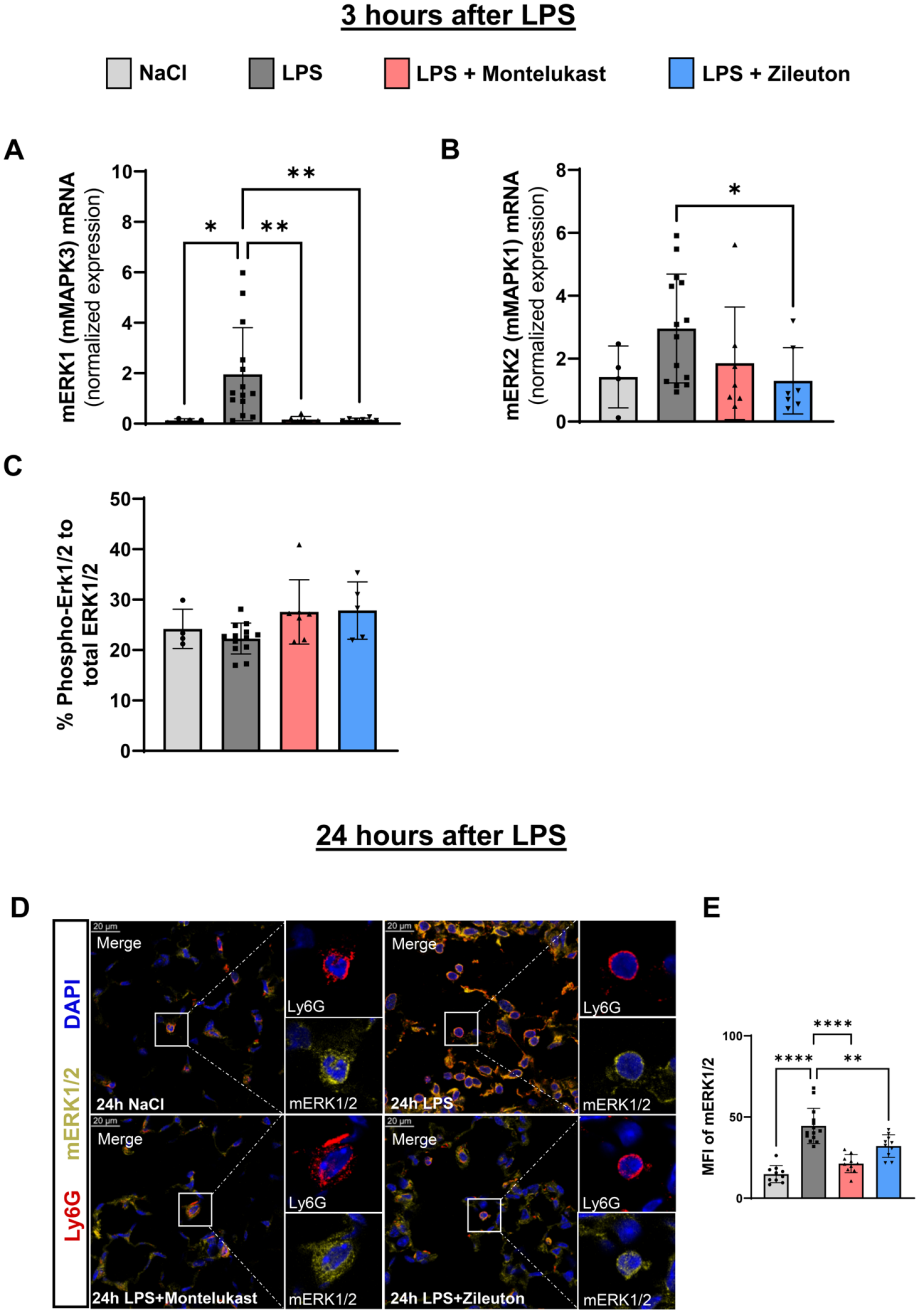
Montelukast and zileuton reduce ERK1/2 signaling in response to LPS. Gene expression of (A) ERK1 (MAPK3) and (B) ERK2 (MAPK1) three hours after LPS inhalation, measured by qPCR. (C) Percentage of Phospho‐ERK1/2 versus total ERK1/2 in lung tissue 3 h after LPS inhalation. (D) Immunofluorescence images showing ERK1/2 protein expression in lung tissue (×63 magnification). (E) Quantification of ERK1/2 MFI in lung sections (*n* = 4 mice per group; 10–14 measurements per group). The bar graphs were labeled according to the following color scheme for the four experimental conditions at the indicated time points: NaCl in light gray, LPS in dark gray, Montelukast in red, and Zileuton in blue. Data are presented as mean ± SD. Statistical analysis: *p < 0.05, **p < 0.01, ****p < 0.0001, using one‐way ANOVA.

Quantitative PCR showed that LPS exposure significantly increased ERK1 and ERK2 mRNA levels, while montelukast and zileuton markedly reduced ERK1 expression, with zileuton also decreasing ERK2 (Figure 6A,B). However, phospho‐ERK1/2 levels remained unchanged. Immunofluorescence analysis confirmed that total ERK1/2 protein levels were elevated in neutrophils following LPS exposure and significantly reduced by both inhibitors (Figure 6C,D).

These findings suggest that montelukast and zileuton may attenuate neutrophil activation and recruitment by suppressing ERK1/2 expression.

## 4 Discussion

In this study, we investigated the therapeutic potential of leukotriene inhibition with montelukast and zileuton in a murine model of LPS‐induced ARDS, focusing on their effects on PMN migration, cytokine release, receptor expression, and ERK1/2 expression. Both drugs significantly reduced PMN infiltration into the bronchoalveolar space; mechanistically, they acted at multiple levels: suppressing adhesion molecule expression, reducing proinflammatory cytokine release, downregulating leukotriene receptors, and attenuating ERK1/2 expression. These findings suggest that leukotriene inhibition disrupts key pathways driving PMN recruitment and activation in acute lung inflammation.

We selected PSGL‐1, L‐selectin, and LFA‐1 as representative adhesion molecules because they coordinate key phases of the multistep neutrophil recruitment process. PSGL‐1 mediates initial tethering and rolling through its interaction with endothelial selectins; L‐selectin regulates early adhesive interactions and neutrophil activation; and LFA‐1 is essential for firm adhesion and subsequent transendothelial migration into inflamed tissues [20, 21, 22]. These molecules were therefore well suited to capture discrete functional changes in neutrophil trafficking in response to leukotriene inhibition. In parallel, ERK1/2 was examined as a downstream effector of leukotriene receptor signaling and a central regulator of neutrophil behavior, including chemotaxis, inflammatory gene transcription, and cytoskeletal dynamics [18]. Notably, ERK1/2 signaling also contributes to the synthesis of leukotrienes themselves, suggesting a feed‐forward loop that may amplify inflammation unless pharmacologically interrupted [19].

These results align with previous studies showing that leukotriene inhibitors reduce neutrophil infiltration in models of ARDS acute lung injury. For example, montelukast has been shown to decrease neutrophil counts in BAL fluid and lung parenchyma following LPS‐induced ARDS lung injury [15]; while zileuton attenuates neutrophil accumulation in bleomycin‐induced lung injury and airway inflammation [26, 27]. Our findings expand upon this by demonstrating that montelukast and zileuton differentially regulate PMN adhesion molecules, specifically PSGL‐1, L‐selectin, and LFA‐1, which are essential for neutrophil transmigration (Figure 2). The observed downregulation of these adhesion molecules may provide a targeted mechanism through which leukotriene inhibitors interfere with PMN migration pathways.

In addition to adhesion regulation, both drugs suppressed the release of key proinflammatory cytokines, including TNF‐α, CXCL2, IL‐1β, and IL‐6, in BAL fluid. Interestingly, CXCL1 levels remained unchanged, despite its similar role as a neutrophil chemoattractant. This discrepancy may reflect differential regulation of cytokine production, as CXCL1 expression may be less dependent on leukotriene signaling and more influenced by alternative pathways such as NF‐κB activation [1, 2]. These findings highlight the selective nature of leukotriene inhibition in modulating cytokine profiles, suggesting that while leukotriene inhibitors broadly dampen inflammation, certain inflammatory signals remain active. The selected 24‐h time point for these analyses corresponds to the peak of neutrophil infiltration and adhesion molecule surface expression following LPS exposure, as previously demonstrated by Reutershan et al. in a murine model of acute lung injury [25]. Neutrophil counts in BAL begin to decline after 48 h, though they do not fully return to baseline within this timeframe. Future studies should investigate whether this selective action preserves essential immune responses while mitigating excessive PMN‐driven inflammation.

Beyond cytokine modulation, montelukast and zileuton downregulated CysLTR1 expression on PMNs, a key receptor involved in leukotriene signaling. Previous studies have shown that inflammatory cytokines upregulate CysLTR1 in conditions such as asthma [6]. By counteracting this upregulation, leukotriene inhibitors not only block receptor‐mediated signaling but may also reduce PMN sensitivity to inflammatory cues. This receptor downregulation provides an additional level of control over neutrophil activation and may contribute to the observed attenuation of inflammatory signaling.

We also found that both inhibitors reduced ERK1/2 expression, a central component of neutrophil activation, migration, and cytokine production [28, 29]. ERK1/2 activation typically occurs downstream of CysLTR1, linking leukotriene signaling to MAPK‐driven inflammatory responses [18, 30]. By downregulating CysLTR1, montelukast and zileuton appear to attenuate ERK1/2 activation, further limiting neutrophil recruitment and excessive inflammatory signaling. This finding suggests that leukotriene inhibition exerts anti‐inflammatory effects not only at the receptor level but also by disrupting downstream signaling pathways that amplify lung inflammation.

Compared to corticosteroids such as dexamethasone, which broadly suppress inflammatory gene expression via glucocorticoid receptor activation [31, 32], leukotriene inhibitors provide a more targeted approach by specifically disrupting leukotriene‐driven inflammation. This selectivity may reduce the risk of systemic immunosuppression, an important consideration in conditions like ARDS where preserving essential immune responses is critical. In addition to leukotriene inhibition and corticosteroids, several emerging molecular pathways offer therapeutic potential in ARDS. Extracellular adenosine signaling is upregulated under hypoxic conditions and exerts anti‐inflammatory effects via A2A and A2B receptors [33]. Pharmacologic strategies to enhance adenosine signaling, such as ENT inhibitors or A2B receptor agonists, could complement leukotriene blockade by stabilizing endothelial integrity and limiting neutrophil recruitment. Similarly, microRNAs such as miR‐21 modulate neutrophil activation and vascular leakage and are being investigated as anti‐inflammatory agents with cell‐type specificity [34, 35]. Although delivery remains a challenge, miRNA‐based therapies could offer more targeted immunomodulation. Hypoxia‐inducible factors (HIFs), particularly HIF‐1α, orchestrate protective genetic programs during acute lung injury, enhancing cellular resilience and promoting repair [36, 37]. Stabilization of HIF signaling could represent another complementary strategy to dampen inflammation and preserve tissue integrity in ARDS. Future studies should evaluate whether combining leukotriene inhibitors with corticosteroids offers a synergistic anti‐inflammatory effect while minimizing immune suppression.

Our study provides novel evidence that montelukast and zileuton mitigate acute lung inflammation by modulating PMN adhesion, cytokine release, leukotriene receptor expression, and ERK1/2 signaling. These findings suggest that leukotriene inhibitors could serve as adjunctive therapies for inflammatory lung conditions such as ARDS, where excessive neutrophil activation exacerbates tissue damage. Future research should explore whether these effects extend to clinical models of ARDS and whether leukotriene inhibitors could be combined with existing anti‐inflammatory treatments to optimize therapeutic outcomes.

## Author Contributions

Anna Biedritzky and Yi Zhang performed animal experiments and analyzed the data. Anika Fuhr, Carolin Kleinmaier, and Jutta Gamper‐Tsigaras supported experiments and analyzed data; Ka‐Lin Heck‐Swain and Kristian‐Christos Ngamsri provided expertise in experimental design and supported data analysis. Franziska Konrad supervised the project and co‐wrote the manuscript; Michael Koeppen designed the study, interpreted data, and wrote the manuscript.

## Conflicts of Interest

The authors declare no conflicts of interest.

## Supporting information


**Data S1:** fsb270934‐sup‐0001‐supinfo.pdf.

## Data Availability

The data that support the findings of this study are available within the article and its Supporting Information. Larger raw datasets underlying Figures 1, 2, 3, 4, 5, 6 and Figure S1,S2 (e.g., raw and processed flow cytometry [FCS] files, ELISA readouts, immunohistochemistry images, and confocal image stacks) are available from the corresponding authors upon reasonable request. Requests should be directed to Franziska M. Konrad (franziska.konrad@rkh-gesundheit.de) or Michael Koeppen (michael.koeppen@med.uni-tuebingen.de).
