# Supplementary material for: Modulation of Neutrophil Recruitment and Inflammatory Signaling in Acute Respiratory Distress Syndrome by Leukotriene Inhibitors Montelukast and Zileuton
Source: FASEB J. 2025 Aug 23;39(16):e70934. doi: 10.1096/fj.202501684R (PMC12374247; doi:10.1096/fj.202501684R)
Supplement: Supplementary file 1 — Data S1: fsb270934‐sup‐0001‐supinfo.pdf. [file FSB2-39-e70934-s001.pdf]

Supplement to:

Modulation of Neutrophil Recruitment and Inflammatory Signaling in Acute Respiratory Distress Syndrome by Leukotriene Inhibitors Montelukast and Zileuton

Anna Biedritzky[1], Yi Zhang[2], Anika Fuhr[1], Carolin Kleinmaier[1], Jutta Gamper-Tsigaras[1], Ka-Lin Heck-Swain[1], Kristian-Christos Ngamsri[1], Franziska Konrad[3], Michael Koeppen[1]

[1] Department of Anesthesiology and Intensive Care Medicine, University Hospital of Tuebingen, Germany
[2] Department of Anesthesiology, Shenzhen Hospital of Southern Medical University, Shenzhen, 518110, Guangdong, China
[3] Klinik für Anästhesiologie, Orthopädische Klinik Markgröningen, Germany

Correspondence to:

Franziska M. Konrad, MD
Klinik für Anästhesiologie
Orthopädische Klinik Markgröningen
Kurt-Lindemann-Weg 10
71706 Markgröningen
Franziska.konrad@rkh-gesundheit.de

OR

Michael Koeppen, MD
Department of Anesthesiology and Intensive Care Medicine
University of Tuebingen
Hoppe-Seyler-Strasse 3
72076 Tuebingen, Germany
Phone: 0049/7071-2986935
Fax: 0049/7071-2986935
E-mail address: michael.koeppen@med.uni-tuebingen.de

Supplementary Figure 1

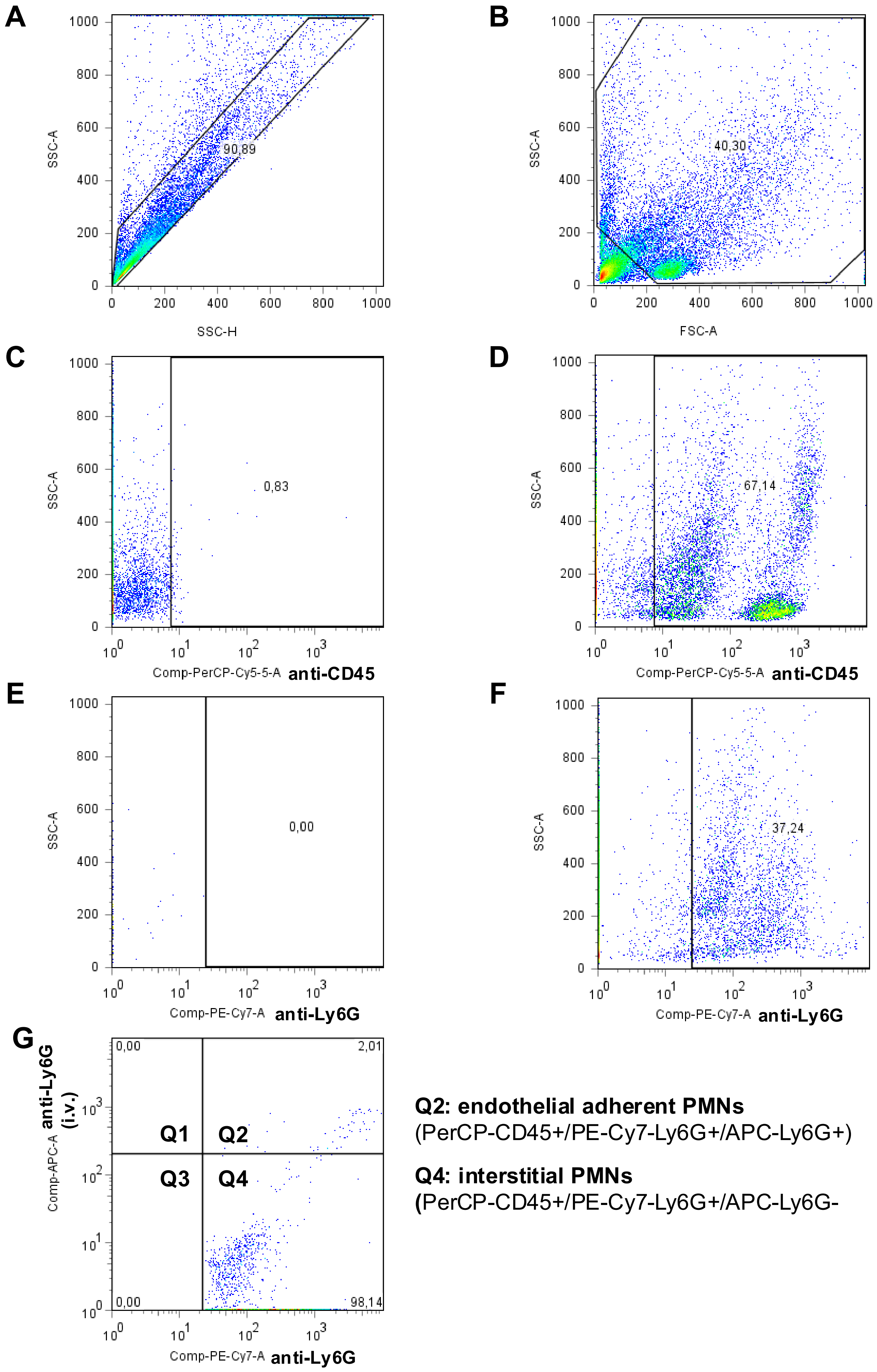

Q2: endothelial adherent PMNs
(PerCP-CD45+/PE-Cy7-Ly6G+/APC-Ly6G+)

Q4: interstitial PMNs
(PerCP-CD45+/PE-Cy7-Ly6G+/APC-Ly6G-

Supplementary Figure 2

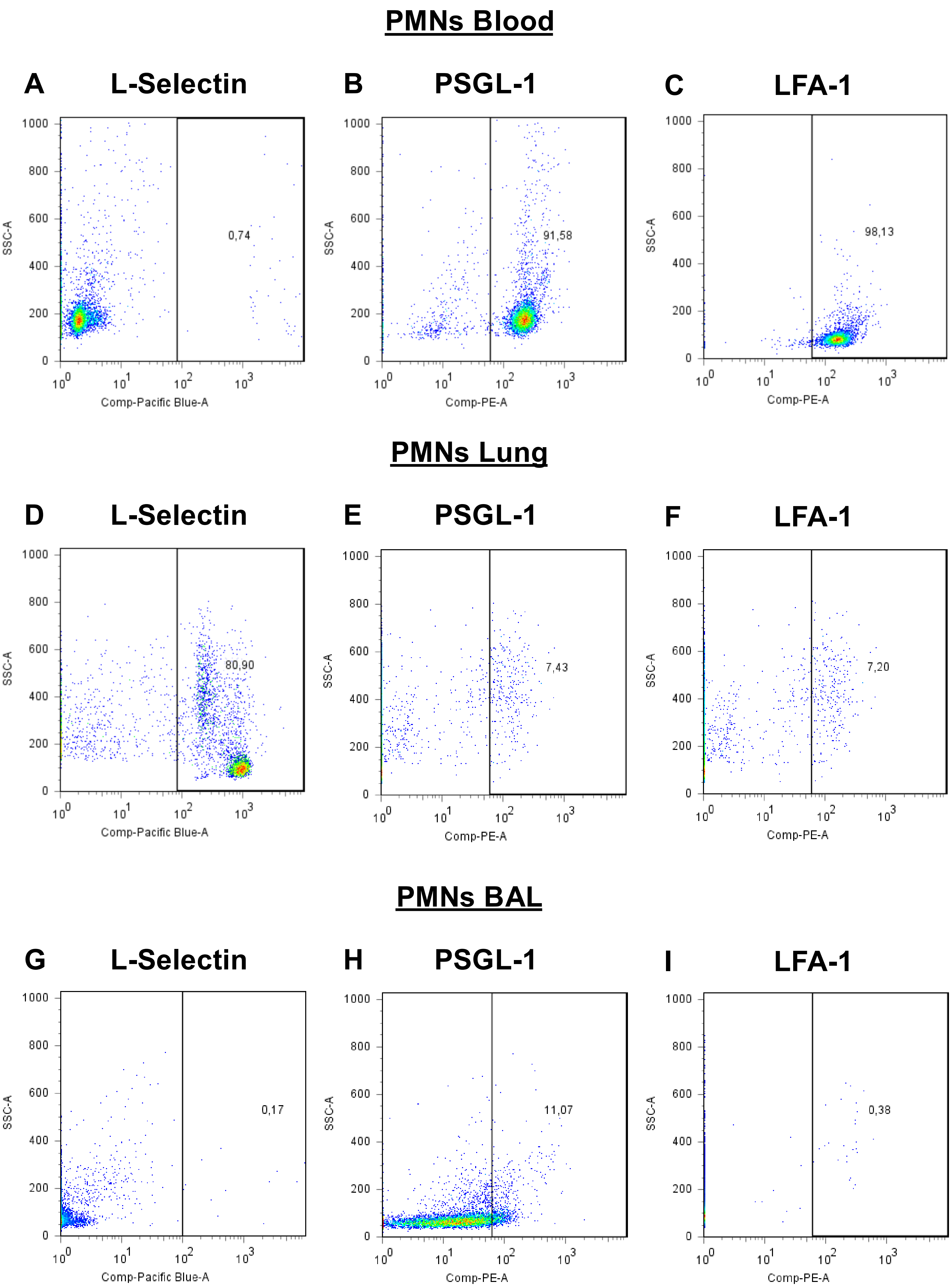

Supplementary Figure 1. Gating strategy for the identification of PMNs.

Gating strategy for flow cytometry-based analysis of PMN distribution across the lung compartments using the example of a representative lung sample. **(A)** Single cell gating of all measured cells. **(B)** Gating of potential leukocytes based on size (FSC) and granularity (SSC). (C) Gating of PerCP-CD45+ leukocytes (unstained sample). **(D)** Gating of PerCP-CD45+ leukocytes (representative sample). **(E)** Gating of PE-Cy7-Ly6G+ PMNs (unstained sample). **(F)** Gating of PE-Cy7-Ly6G+ PMNs (representative sample). **(G)** Quadrant gate of PE-Cy7 and APC to identify PerCP-CD45+/PE-Cy7-Ly6G+/APC-Ly6G+ adherent PMNs (Q2) and PerCP-CD45+/PE-Cy7-Ly6G+/APC-Ly6G- interstitial PMNs (Q4).

Supplementary Figure 2. Representative flow plots for the adhesion molecules L-selectin, PSGL-1, and LFA-1 on PMNs in blood, lung and BAL

Representative flow plots of the adhesion molecules (A) L-Selectin, (B) PSGL-1), and (C) LFA-1 on PMNs in blood, (D) L-Selectin, (E) PSGL-1, and (F) LFA-1 on PMNs in lung, and (G) L-Selectin, (H) PSGL-1, and (I) LFA-1 on intra-alveolar PMNs.
